# Using Mendelian randomization to understand and develop treatments for neurodegenerative disease

**DOI:** 10.1093/braincomms/fcaa031

**Published:** 2020-03-20

**Authors:** Catherine S Storm, Demis A Kia, Mona Almramhi, Nicholas W Wood

**Affiliations:** Department of Clinical and Movement Neurosciences, University College London Queen Square Institute of Neurology, London, UK

**Keywords:** Mendelian randomization, neurodegeneration, genetics

## Abstract

Common neurodegenerative diseases are thought to arise from a combination of environmental and genetic exposures. Mendelian randomization is a powerful way to leverage existing genetic data to investigate causal relationships between risk factors and disease. In recent years, Mendelian randomization has gathered considerable traction in neurodegenerative disease research, providing valuable insights into the aetiology of these conditions. This review aims to evaluate the impact of Mendelian randomization studies on translational medicine for neurodegenerative diseases, highlighting the advances made and challenges faced. We will first describe the fundamental principles and limitations of Mendelian randomization and then discuss the lessons from Mendelian randomization studies of environmental risk factors for neurodegeneration. We will illustrate how Mendelian randomization projects have used novel resources to study molecular pathways of neurodegenerative disease and discuss the emerging role of Mendelian randomization in drug development. Finally, we will conclude with our view of the future of Mendelian randomization in these conditions, underscoring unanswered questions in this field.

## Teaser

Mendelian randomization uses genetic data to examine causal relationships. Storm *et al.* review the insights gained from Mendelian randomization studies about environmental risk factors, molecular mechanisms and drug development for neurodegenerative diseases. The authors critically evaluate novel approaches in this field and explore the challenges going forward.

## Graphical Abstract

**Graphical Abstract:**
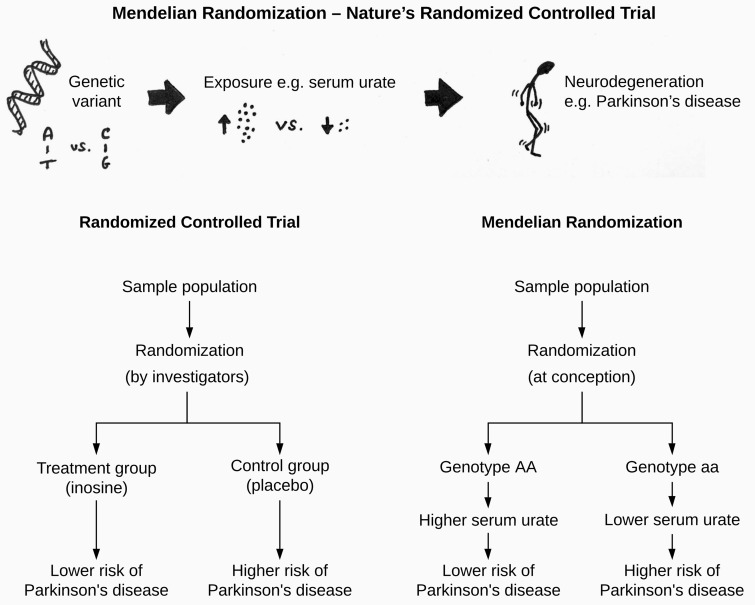

## Introduction

Constructing a thorough understanding of neurodegenerative disease aetiology is a multidisciplinary enterprise. Observational studies have uncovered many environmental risk factors, yet spurious associations may arise if confounding variables influence both exposure and disease. There may also be reverse causation, where the disease causes the exposure. Randomized controlled trials (RCTs) are less affected by such issues; however, large-scale RCTs are prohibitively expensive.

The genetic determinants of neurodegenerative disease have been explored in genome-wide association studies (GWASs) (IMSGC and WTCCC2, 2011; IMSGC, 2013, 2019; GeM-HD Consortium, 2015; van Rheenen *et al.*, 2016; Chang *et al.*, 2017; Jansen *et al.*, 2019; Nalls *et al.*, 2019). GWASs are a tremendous resource for studying molecular pathways and drug targets, and medications with genetic support may be twice as likely to proceed from Phase I to approval (Nelson *et al.*, 2015).

Mendelian randomization (MR) is a powerful method to investigate the interplay between genetic and environmental disease risks, enabling a more robust understanding of pathogenesis. MR has recently gathered considerable traction in neurodegenerative disease research, and this review aims to evaluate the impact of MR studies on translational medicine for these conditions. We will

- explain the principles and limitations of MR,
- discuss lessons from MR studies of environmental risk factors for neurodegeneration,
- describe how MR can provide insight about molecular disease pathways,
- address the emerging role of MR in drug development and
- conclude with the future directions and limitations of MR in neurodegenerative diseases.

## Search strategy

The PubMed and EMBASE databases were searched for key words such as ‘Mendelian randomization’, ‘neuro*’, ‘Parkinson*’, ‘Alzheimer*’, ‘dementia’, ‘Huntington*’ and ‘multiple sclerosis’ to identify MR studies published before 23 July 2019, with no language restrictions. Further articles were located through the references of these articles. Studies were assessed for relevance based on titles, abstracts and full texts.

## What is Mendelian randomization?

MR builds on the principle that genetic variants mimic exposure to environmental risk factors (Katan, 1986; Davey Smith and Ebrahim, 2003). Since GWASs have identified single-nucleotide polymorphisms (SNPs) associated with many phenotypic traits, MR can be used to interrogate questions such as: is higher education protective against Alzheimer’s disease?

**Figure 1:**
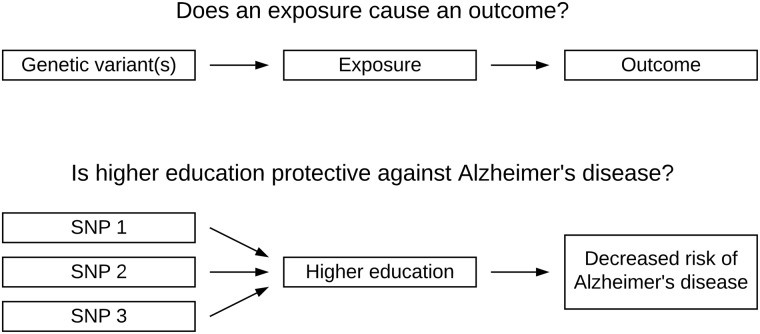
**Mendelian randomization as a concept.** Mendelian randomization investigates if an exposure causes an outcome by using genetic variants that are strongly associated with the risk factor of interest. For example, genetic variants linked to education can be used to infer whether education has an effect Alzheimer’s disease risk.

Methodologically, SNPs that are associated with an exposure of interest (e.g. education) are used as the so-called ‘instrumental variable’ or ‘genetic instrument’. The association between the same genetic variants and an outcome (e.g. Alzheimer’s disease) is then calculated (Fig. 1). The SNP-exposure and SNP-outcome associations are combined to infer whether the exposure causes the outcome.

MR overcomes many limitations of observational studies and RCTs. Since the inheritance of a genetic variant is independent of other traits (Mendel’s law of independent assortment), any confounders should be equally common in people with different genotypes. MR is less susceptible to reverse causation, because genotypes are determined at conception and not modifiable by disease. Thanks to openly available GWAS data, MR is much cheaper and quicker than a large-scale observational study or RCT. There are nonetheless key limitations of MR, which are explained in Box 1.

## Lessons from MR studies of environmental risk factors for neurodegeneration

### Confirming findings from epidemiology—Alzheimer’s disease and education

Observational data suggest that one in five Alzheimer’s disease cases may be attributable to low educational attainment (Norton *et al.*, 2014). This association may be confounded by, e.g. socioeconomic status (Raghavan *et al.*, 2019), providing an opportunity for MR studies to shed light.

An MR study by Østergaard *et al.* found no link between genetically predicted university completion or more years of education and Alzheimer’s disease risk (Østergaard *et al.*, 2015), whereas another MR project suggested that these risk factors may be protective (Larsson *et al.*, 2017). These conflicting findings can be explained by understanding that the power of an MR study depends on how well the genetic variants predict the exposure. The SNP used by Østergaard and colleagues explained 0.022% of variation in years in education (Rietveld *et al*., 2013), and recent GWASs capture up to 13% and 5.2% of variability in educational attainment and intelligence, respectively (Lee *et al.*, 2018; Savage *et al.*, 2018).

Using the most recent GWASs, an MR study found a decreased Alzheimer’s disease risk with more years in education (Raghavan *et al.*, 2019). There was a small population overlap between the GWASs for education and Alzheimer’s disease, which can bias the MR result (Pierce and Burgess, 2013). Nevertheless, three similarly powered MR analyses found similar results (Anderson *et al.*, 2020; Savage *et al.*, 2018; Jansen *et al.*, 2019). Taken together, genetic evidence indicates that low educational attainment plays a causal role in Alzheimer’s disease.

### Relationships between diseases—Alzheimer’s disease, Parkinson’s disease and rheumatoid arthritis

Alzheimer’s disease and Parkinson’s disease share clinical and pathological features (Hamilton, 2000; Hanagasi and Emre, 2005; Kalia and Lang, 2015); however, there is little overlap in their genetic risk (The Brainstorm Consortium, 2018). An MR study has provided further evidence, showing that genetically predicted Parkinson’s disease does not affect Alzheimer’s disease risk (Han *et al.*, 2018). The authors suggested that an SNP in the alpha-synuclein gene *SNCA* (a risk locus for Parkinson's Disease) was protective for Alzheimer’s disease, but only when using one statistical method. There are many methods to calculate the MR effect, and it is advised to use several approaches with different assumptions to assess the robustness of results (Haycock *et al.*, 2016; Burgess *et al.*, 2017; Slob and Burgess, 2020). If only one method yields a significant result, this should be interpreted with caution.

Furthermore, observational studies suggest that rheumatoid arthritis (an auto-immune inflammatory condition of joints) is inversely associated with Alzheimer’s disease (Smolen *et al.*, 2016). A 2017 MR study found no causal link between these conditions (Policicchio *et al.*, 2017). A different MR study directly probed reverse causation, showing that genetic risk of Alzheimer’s disease does not affect rheumatoid arthritis risk either (Cai *et al.*, 2018). A third MR study found that genetically predicted rheumatoid arthritis raised Alzheimer’s disease risk (Bae and Lee, 2018), but the result lost significance when one SNP was removed from the analysis. This suggests that the MR result is driven or biased by this SNP and that this outlier may affect disease risk through an alternative mechanism, rather than through rheumatoid arthritis (Burgess *et al.*, 2017).

### Challenges to interpreting MR studies—Parkinson’s disease and body mass index

The clinical implications of MR results are not always straightforward, as is illustrated by an MR study about body mass index (BMI) and Parkinson’s disease. Observational evidence has been conflicting (Wang *et al.*, 2015; Wills *et al.*, 2016; Roos *et al.*, 2018), and an MR study found that genetically higher BMI is protective against Parkinson’s disease (Noyce *et al.*, 2017). Current MR methods detect *whether* there is a causal relationship, rather than the *magnitude* thereof, so it is not clear how much of a BMI change would be needed to influence Parkinson’s disease risk. Given that a raised BMI is a well-established risk factor for cardiovascular disease (Benjamin *et al.*, 2019), promoting a raised BMI for the sake of Parkinson’s disease protection produces a challenge.

### Environmental risk factors and ethnicity—amyotrophic lateral sclerosis and blood lipids

MR can also be used to explore how disease aetiology differs between ethnic groups. The incidence of amyotrophic lateral sclerosis (ALS) varies between continents such as Europe and Asia but seems homogenous between Europe, North America and New Zealand (Marin *et al.*, 2017). Such patterns may reflect differences in genetic and/or environmental risks.

Reviews suggest that hyperlipidaemia may be protective for ALS prognosis and risk (Jawaid *et al.*, 2018). An MR study assessed whether low-density lipoprotein cholesterol, high-density lipoprotein cholesterol, total cholesterol and triglyceride levels affect ALS risk, using different GWAS data for Europeans and East Asians (Zeng and Zhou, 2018). The authors found that raised low-density lipoprotein cholesterol was significantly associated with increased ALS risk in Europeans, which is in line with another MR study in Europeans (Bandres-Ciga *et al.*, 2019*a*).

Zeng and Zhou found similar results in an East Asian cohort, but at nominal significance only (Zeng and Zhou, 2018). This population was smaller than the European cohort, so it is unclear whether there is a true biological difference or a lack of power in the East Asian group. Moreover, it has been proposed that observational studies may be confounded by raised BMI, which has been linked to slower ALS progression (Jawaid *et al.*, 2018). Using MR, Zeng et al. showed that genetically predicted BMI is not causally associated with ALS in Europeans nor East Asians (Zeng *et al.,* 2019).

Well-powered GWASs are key to a successful MR, and large GWASs remain mostly limited to European populations (Popejoy and Fullerton, 2016; Sirugo *et al.*, 2019). As such, ethnically diverse GWASs and MR studies are crucial before we can generalize MR results to people of different ancestries.

## Using MR to study molecular mechanisms of neurodegenerative disease

MR has recently been extended to molecular disease mechanisms using ‘summary-based MR’, which combines GWAS and gene expression data (Zhu *et al.*, 2016). This is the same as classical MR, except the exposure is the expression of a gene. Expression quantitative trait loci (QTL) are used, which are genetic variants associated with different expression levels of a gene. SNPs associated with gene methylation QTL or protein QTL levels can also be studied using this method.

This approach can identify new genes that may be relevant to disease aetiology. One summary-based MR study used expression QTL associated with the expression of 5366 genes in peripheral blood and data from an ALS GWAS, finding five genes whose expression levels predict ALS risk, none of which were detected by the GWAS (Du *et al.*, 2018). Summary-based MR can also be used to interrogate disease pathways. One study used genes related to endosomal membrane trafficking pathways and showed that expression and methylation of these genes predicted Parkinson’s disease risk (Bandres-Ciga *et al.*, 2019*c*).

By using the MR approach, these studies provide insight into the ‘causal’ molecular biology of neurodegeneration, and summary-based MR and a derivative thereof (Zhu *et al.*, 2018) have now formed part of several GWASs for neurodegenerative disease (Benyamin *et al.*, 2017; Jansen *et al.*, 2019; Nalls *et al.*, 2019).

## MR in drug development—nature’s randomized controlled trial

It currently takes approximately a decade and $2.6 billion for one drug to proceed from initial testing in humans to licencing (DiMasi *et al.*, 2016), and ∼90% of drugs in Phase I clinical trials are never launched, mostly due to insufficient efficacy and safety (Harrison, 2016; Smietana *et al.*, 2016).

**Figure 2:**
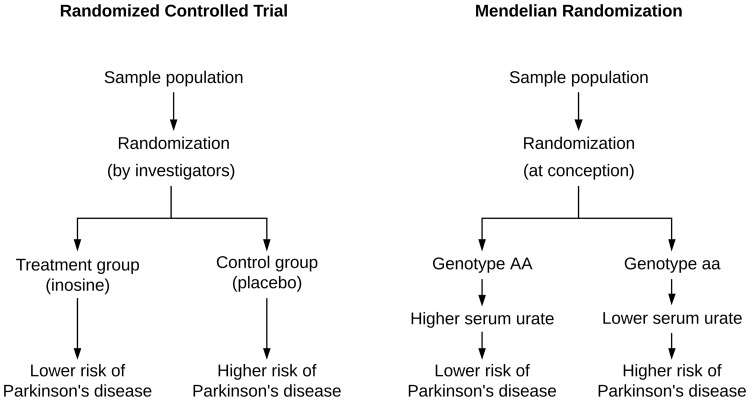
**Mendelian randomization is analogous to a placebo-controlled randomized controlled trial.** For example, genetic variants associated with higher serum urate levels mimic lifelong exposure to a urate-raising drug.

MR provides an exciting opportunity, because it can imitate an RCT. The underlying principle is that genetic variants associated with different concentrations of a drug target act in the same way the corresponding drug does (Evans and Davey Smith, 2015). The SNPs essentially mimic lifelong exposure to low levels of a drug, and the random allocation of genes at birth is similar to randomization in an RCT. In addition, patients do not typically know their genotype, so MR studies are effectively blinded. Figure 2 visualizes the analogy between MR and an RCT, using serum urate and Parkinson’s disease as an example.

MR offers a chance to prioritize drug targets using human evidence early in the drug development pipeline. We can also explore repurposing opportunities for already-licenced drugs, which have passed safety assessment and could reach patients sooner and at a lower cost (Evans and Davey Smith, 2015). MR has already been applied in drug development for neurodegenerative disease, and below we discuss several promising examples.

### Parkinson’s disease and urate

Epidemiological evidence indicates that higher serum urate may lower Parkinson’s disease risk (Gao *et al.*, 2016; Wen *et al.*, 2017). Four small MR studies have found a protective role of raised serum urate on Parkinson’s disease risk (Gao *et al.*, 2013; González-Aramburu *et al.*, 2013), age of onset (Facheris *et al.*, 2011) and progression to disability requiring dopaminergic treatment at 1 year (Simon *et al.*, 2014). More recently, two considerably larger MR studies found no significant association between plasma urate and Parkinson’s disease risk (Kia *et al.*, 2018; Kobylecki *et al.*, 2018).

This discrepancy may lie in the chosen instrumental variable and sample sizes. Genetically determined urate levels are largely defined by variation in *SLC2A9* and *ABCG2*, which together explain ∼3.4% of serum urate concentration (Köttgen *et al.*, 2013). Most of the early MR studies of urate used SNPs in *SLC2A9* only (Facheris *et al.*, 2011; Gao *et al.*, 2013, Simon *et al.*, 2014). Kia *et al.* used GWAS-identified SNPs that explain 7% of urate variability and a two-sample MR design combining two large-scale GWAS studies, both of which improve statistical power (Kia *et al.*, 2018).

In 2018, a clinical trial studying the urate-raising drug inosine as a treatment for Parkinson’s disease was terminated early, because it was deemed ‘unable to show that inosine slows Parkinson’s progression’ (https://www.michaeljfox.org/news/parkinsons-inosine-trial-ending-early, last accessed 9 April 2020). This potently shows how well-powered MR studies can predict the likely efficacy of a drug.

### Alzheimer’s disease and blood pressure

Observational studies suggest that hypertension is a risk factor for Alzheimer’s disease (Norton *et al.*, 2014; Livingston *et al.*, 2017) and that antihypertensive use may be protective (Larsson and Markus, 2018), creating a potential drug repurposing opportunity.

One MR study opposed this evidence, finding that genetically predicted higher systolic blood pressure may decrease Alzheimer’s disease risk (Østergaard *et al.*, 2015). A more recent MR study used SNPs associated with the expression of 12 antihypertensive drug targets and systolic blood pressure (Walker *et al.*, 2019). Only the proxy for angiotensin-converting enzyme inhibitors was associated with Alzheimer’s disease risk.

Furthermore, another MR study has shown that acetylcholinesterase expression may be causally associated with raised blood pressure (Richardson *et al.*, 2020). *ACHE* encodes the target for acetylcholinesterase inhibitors such as donepezil and galantamine, which are licenced treatments for Alzheimer’s disease (Lane *et al.*, 2018).

MR can also be used to untangle on-target and off-target drug effects. Walker *et al.* postulated that angiotensin converting enzyme inhibitors may act through a different mechanism, since no link was found between Alzheimer’s disease risk and systolic blood pressure or other antihypertensives (Walker *et al.*, 2019). This principle has been illustrated in cardiometabolic disease, building on observations that statin therapy is associated with an increased risk of type 2 diabetes mellitus (Sattar *et al.*, 2009). Using SNPs in the HMG-CoA reductase gene *HMGCR*, which encodes the target for statins, an MR study showed that the raised diabetes risk is an on-target effect (Swerdlow *et al.*, 2015). Likewise, if MR evidence suggests that a side effect is an off-target effect, this could encourage the development of a drug with more specificity (Evans and Davey Smith, 2015).

### Multiple sclerosis and vitamin D

Multiple sclerosis prevalence rises with latitude on both sides of the equator (Ascherio and Schwarzschild, 2016; Sintzel *et al.*, 2018), and migration studies suggest that multiple sclerosis risk is heavily influenced by environmental factors (Berg-Hansen and Celius, 2015; Ascherio and Munger, 2016; Dobson and Giovannoni, 2019). This is thought to occur because ultraviolet light stimulates vitamin D production in the skin, and vitamin D levels are inversely correlated with multiple sclerosis risk (Sintzel *et al.*, 2018).

MR studies show that genetically predicted lower vitamin D may increase the risk of multiple sclerosis (Mokry *et al.*, 2015; Rhead *et al.*, 2016) and paediatric-onset multiple sclerosis (Gianfrancesco *et al.*, 2017). The paediatric study used SNPs from a GWAS in adults, so these SNPs may not reflect vitamin D status in children. In addition, most control subjects were adults. Control subjects are traditionally age-matched, yet it is possible that paediatric controls may develop multiple sclerosis later in life, whereas adult controls are definite ‘negatives’ for paediatric multiple sclerosis. Furthermore, present-day MR assumes a linear relationship, so it is unclear if vitamin D is always protective, or for example only in clinically deficient individuals.

### Disease risk as an outcome—Huntington’s disease and telomeres

An important limitation of MR in drug development is that most GWAS datasets pertain to disease *risk* rather than *progression*. In other words, most MR studies to date predict whether an intervention could *prevent* disease, rather than slow or hinder its progression. For example, although MR studies show an inverse relationship between vitamin D levels and multiple sclerosis risk, no significant association has been found with multiple sclerosis severity nor age at onset (Rhead *et al.*, 2016).

An exception to this trend pertains to Huntington’s disease, a neurodegenerative condition caused by a trinucleotide (cytosine-adenine-guanine) repeat expansion in the huntingtin gene (Ross *et al.*, 2014). The trinucleotide repeat length determines some but not all variability in age at motor symptom onset (GeM-HD Consortium, 2015), and an MR study suggests that longer telomeres (repetitive DNA sequences at the end of chromosomes) may delay Huntington’s disease onset (Aziz and Weydt, 2018). These SNPs are associated with telomere length in leukocytes, which are correlated with that in neurons (Zhan *et al.*, 2015; Aziz and Weydt, 2018). Generally, an MR instrument and exposure do not have to be functionally linked and they must only be strongly correlated, so that the SNP(s) reliably represents the exposure.

## Future directions and outstanding questions

The expanding popularity of MR has facilitated its critical evaluation and steady improvement (Burgess *et al.*, 2015, 2018; Holmes *et al.*, 2017; Hemani *et al.*, 2018), and initiatives such as the UK Biobank, Million Veterans Program and 23andMe (https://research.23andme.com/, last accessed 9 April 2020) provide genetic data for a wealth of phenotypic traits (Gaziano *et al.*, 2016; Bycroft *et al.*, 2018). In addition, guidelines have been produced to standardize the reporting quality of MR studies (Burgess *et al*., 2019, Davey Smith *et al.*, 2019).

These developments make MR increasingly accessible, enabling ‘high-throughput’ projects such as an online platform collating over 5000 GWASs to facilitate MR for Parkinson’s disease (Noyce *et al.*, 2019). Nonetheless, it remains crucial to perform MR studies with a reasoned approach, carefully tailored to the research question (Burgess and Davey Smith, 2019). Together with ethnically diverse data and GWASs for progression (Blauwendraat *et al.*, 2019; Iwaki *et al.*, 2019), these resources will expedite MR for neurodegenerative disease prevention and drug development.

With regard to molecular-level MR, it remains unclear whether eQTLs are the best tool. Since most drugs act on proteins, it is perhaps more suitable to use pQTLs, i.e. SNPs associated with different protein levels rather than gene expression. pQTL studies to date have measured protein levels in blood only (Emilsson *et al.*, 2018; Sun *et al.*, 2018; Yao *et al.*, 2018), which may differ from the central nervous system. On the other hand, if MR evidence shows that blood protein levels affect neurodegenerative disease, a corresponding drug may not need to cross the blood–brain barrier to exert a therapeutic effect.

Tissue diversity has been more thoroughly explored for eQTL data (Gamazon *et al.*, 2018; Taylor *et al.*, 2019; Richardson *et al.*, 2020). For example, an MR study found that *FBN2* expression in heart tissue was linked to diastolic blood pressure, whereas *FBN2* expression in lung tissue was linked to forced vital capacity (Richardson *et al.*, 2020). Such evidence suggests that biologically relevant tissues may be crucial for molecular-level MR projects. The sample sizes of tissue-diverse studies are small (Gamazon *et al.*, 2018; Võsa *et al.*, 2018), so the importance of tissue specificity remains uncertain. Finally, QTL data are notably not functional and these SNPs may not accurately reflect protein activity.

## Conclusion

If we view disease aetiology as a combination of genetics and environment, MR provides an invaluable meeting point. MR has yielded many fruitful results and lessons for neurodegeneration, propelling our understanding about environmental risk factors and molecular pathophysiology. This engenders an advantageous evidence base for public health interventions to prevent disease. We eagerly anticipate further GWASs with progression outcomes and ethnically diverse populations, as well as tissue-specific QTL data, which may mitigate the challenges to translating MR results to the clinic. As these resources continue to grow, MR becomes an ideal tool for drug development, able to prioritize medications at an early stage and ascertain molecular mechanisms of action. We encourage carefully designed, standardized use of this exciting technique, boosting the number of success stories.

## Funding

C.S.S. is funded by Rosetrees Trust, John Black Charitable Foundation and the University College London MB PhD Programme. D.A.K. is supported by an MB PhD Award from the International Journal of Experimental Pathology. M.A. is funded by the Faculty of Applied Medical Sciences, King Abdulaziz University, Jeddah, Saudi Arabia. N.W.W. is a National Institute for Health Research senior investigator and receives support from the European Union Joint Programme—Neurodegenerative Disease Research Medical Research Council Comprehensive Unbiased Risk factor Assessment for Genetics and Environment in Parkinson’s disease. N.W.W. is also supported by the National Institute for Health Research University College London Hospitals Biomedical Research Centre.

## Competing interests

The authors report no competing interests.

**Box 1** MR considerations and limitations

MR operates under three core assumptions, stating that

- the genetic variant(s) must be associated with the exposure;
- the genetic variant(s) must ‘not’ be associated with any confounders; and
- the genetic variant(s) must ‘not’ be associated directly with the outcome.

The first assumption can be addressed by selecting genetic variants strongly associated with the exposure, e.g. at genome-wide significance (*P* < 5 × 10^−8^). Nevertheless, GWAS-identified SNPs typically have small effect sizes; for example 97 genetic loci account for ∼2.7% of variability in BMI (Locke *et al.*, 2015). Weak instruments limit statistical power of an MR study and can bias the final result (Pierce and Burgess, 2013).

Several SNPs can be combined to mimic one exposure (Haycock *et al.*, 2016; Hemani *et al.*, 2018); e.g. two SNPs represent 2.7% of plasma urate, whereas 26 SNPs explain 7% of urate variance (Kia *et al.*, 2018; Kobylecki *et al.*, 2018). Large study population sizes can also improve statistical power (Haycock *et al.*, 2016), and the exposure and outcome do not have to be measured in the same population. Data from two independent GWASs can therefore be combined using ‘two-sample MR’, as long as the two populations are of the same ancestry.

The latter two core assumptions are violated by genetic pleiotropy, where a genetic locus influences more than one trait. This means that an SNP may affect the outcome through a pathway that does not involve the exposure. Many different methods have been developed to allow for some pleiotropy (Burgess *et al.*, 2015, 2019; Holmes *et al.*, 2017; Hemani *et al.*, 2018).

Some limitations particularly apply to MR in drug development. Most GWASs pertain to disease *risk*, rather than *progression*, and MR instruments mimic lifelong, low-dose exposure to a risk factor or drug. This is useful when studying preventative interventions or public health policies, but perhaps less so when predicting the outcome of clinical trials, which typically last a few years only and measure progression. Indeed, there is evidence that MR may overestimate the effect seen in clinical trials (Burgess *et al.*, 2012; Ference *et al.*, 2012).

In addition, there may not be genetic data available to represent a drug. One could use a proxy measure, such as blood lipids to mimic statins rather than levels of the molecular target (Walker *et al.*, 2017). Such proxies cannot detect effects through unknown mechanisms, and SNPs associated with specific gene expression or protein levels may be more suitable (Gamazon *et al.*, 2018; Sun *et al.*, 2018; Võsa *et al.*, 2018; Yao *et al.*, 2018; Zhernakova *et al.*, 2018).

For further reading on MR methods and limitations, we direct readers to the references (Evans and Davey Smith, 2015; Holmes *et al.*, 2017; Davies *et al.*, 2018, Bandres-Ciga *et al.*, 2019*b*).

## Abbreviations

ALS =: amyotrophic lateral sclerosis
BMI =: body mass index
GWAS =: genome-wide association study
MR =: Mendelian randomization
QTL =: quantitative trait locus
RCT =: randomized controlled trial
SNP =: single-nucleotide polymorphism
